# Reply to “Exploring Bacteria‐Based Cancer Immunotherapy”—Comment on “Discovery of Intratumoral Oncolytic Bacteria Toward Targeted Anticancer Theranostics”

**DOI:** 10.1002/advs.202511325

**Published:** 2025-07-14

**Authors:** Eijiro Miyako

**Affiliations:** ^1^ Graduate School of Advanced Science and Technology Japan Advanced Institute of Science and Technology 1‐1 Asahidai Nomi Ishikawa 923‐1292 Japan

**Keywords:** bacterial theranostics, cancer immunotherapy, intratumoral bacteria, microbiome

## Abstract

This manuscript is a formal response to the commentary by A/Prof. Dr. Chen‐xi Li and colleagues regarding our previous publication in Advanced Science. We clarify the rationale, methods, and outcomes of our original study and highlight ongoing work in the field of bacterial cancer immunotherapy.

We sincerely thank the authors of the Commentary for their deep interest in our recent publication and for their thoughtful perspectives on tumor‐associated microbiota and bacterial immunotherapy. Their recognition of the novelty and therapeutic potential of our work is deeply appreciated. The authors raised several important points, including the rationale behind selecting *Proteus mirabilis* (PM), the identification and tumor‐targeting properties of *Rhodopseudomonas palustris* (RP), and the use of subcutaneous rather than orthotopic models. These are critical issues for advancing the field of bacterial cancer therapy, and we welcome the opportunity to address them.

Our study began with a straightforward hypothesis: that photosynthetic bacteria might exhibit tumor‐homing capabilities and could be harnessed for cancer theranostics–‐as initially explored in our previous publication in *Nano Today* ^[^ ^1^ ^]^ RP, a purple photosynthetic bacterium obtained from a microbial culture bank, was selected based on its optical and biocompatible properties. However, during the course of isolating RP from tumors following systemic administration, we consistently observed unexpectedly potent tumor colonization and regression.

Upon closer investigation, including detailed microbiological and genomic analyses, we discovered that the bacterial population originally believed to be solely RP was in fact a mixed consortium. It contained a second, previously unnoticed intratumoral species—*Proteus mirabilis* (PM)—which had co‐existed with RP from the beginning. This accidental co‐cultivation turned out to be pivotal.

Subsequent systematic screening—detailed in our publications in *Advanced Science* ^[^ ^2^ ^]^ and *Biomedicine & Pharmacotherapy* ^[^ ^3^ ^]^—demonstrated that PM outperformed other intratumoral bacterial isolates in both colonization and oncolytic activity. Although PM was not part of the original experimental design, it emerged as a highly potent component of the microbial consortium. The methodologies for bacterial isolation, cultivation, and comparative evaluation are fully described in these reports.

Analysis of the originally mixed bacterial culture revealed that PM and RP consistently coexisted at a ratio of approximately 3:97, possibly due to intrinsic metabolic interactions such as cysteine dependency. Interestingly, this naturally occurring ratio was found to correspond to the condition under which maximal antitumor efficacy was observed across multiple syngeneic tumor models following intravenous administration.

In our *Advanced Science* article,^[^ ^2^ ^]^ we termed this unique bacterial pairing “AUN” and reported its foundational tumor‐colonizing and oncolytic characteristics. In our follow‐up study—now in press at *Nature Biomedical Engineering* ^[^ ^4^ ^]^—we further elucidated AUN's underlying mechanisms and demonstrated its therapeutic potential in immunocompromised settings. AUN, composed of PM and RP at a 3:97 ratio, induced complete tumor regression and prolonged survival even in immunocompromised mice, independent of cytotoxic immune cell infiltration. Mechanistic studies revealed that its efficacy was driven by a combination of tumor vascular disruption, oncometabolite‐triggered bacterial transformation, and dynamic intratumoral population shifts—resulting in highly selective tumor oncolysis.

Notably, genome sequencing of the tumor‐isolated PM strain (A‐gyo) revealed it to be genetically distinct from standard laboratory PM strains, including deletions in several genes associated with pathogenicity. As described in our forthcoming *Nature Biomedical Engineering* publication, this suggests a naturally selected or tumor‐adapted attenuation of virulence, potentially contributing to its tumor specificity and favorable safety profile. These findings raise exciting possibilities regarding in situ microbial evolution within the tumor microenvironment.

Regarding model selection, we agree that orthotopic models offer superior physiological relevance, especially in the context of immunotherapy research. While subcutaneous models were used for initial screening due to their reproducibility and accessibility, our subsequent investigations—as described in our forthcoming *Nature Biomedical Engineering* publication—include studies using an orthotopic pancreatic cancer model, in which the AUN consortium demonstrated similarly potent antitumor efficacy. These results support the clinical translatability of our findings in anatomically relevant settings.

In parallel with our mechanistic studies, we have also explored engineering strategies to further enhance the therapeutic efficacy of the AUN consortium. In Chemical Engineering Journal,^[^ ^5^ ^]^ we demonstrate that simple scaffold‐mediated modulation of the bacterial culturing significantly augments the antitumor activity of AUN. Specifically, a polydimethylsiloxane‐based porous scaffold incorporating photoactivatable TiO₂ was shown to enhance microbial function and therapeutic synergy in both syngeneic and orthotopic models of triple‐negative breast cancer. Importantly, this approach leverages the unique metabolic and functional adaptability of AUN in response to bacterial microenvironmental cues, including light and reactive oxygen species diffusion. Moreover, initial studies in canine models showed favorable tolerability, highlighting the translational promise of this platform. These findings not only underscore the robustness and adaptability of the AUN system but also suggest new avenues for combining microbial therapy with material engineering for precision cancer treatment.

Finally, we fully agree on the importance of rigorous bacterial identification. Our recent and ongoing work employs 16S rRNA sequencing, PCR‐based assays, and immunohistochemistry to confirm the presence and distribution of both PM and RP in tissues. These multimodal techniques have enabled high‐confidence verification of tumor‐specific colonization while minimizing the likelihood of false positives.

In summary, what began as a straightforward investigation into photosynthetic bacteria for cancer theranostics unexpectedly led to the discovery of a highly synergistic bacterial consortium. First reported as “AUN” in *Advanced Science*,^[^ ^2^ ^]^ and subsequently mechanistically dissected in our follow‐up study,^[^ ^4^ ^]^ this platform has evolved into a promising approach for microbiome‐based cancer therapeutics. We are grateful for the opportunity to elaborate on our work and to contribute to the ongoing advancement of tumor microbiome–based therapeutic strategies.

## Conflict of Interest

The authors declare no conflict of interest.
